# Simultaneous Coinfections with West Nile Virus and Usutu Virus in *Culex pipiens* and *Aedes vexans* Mosquitoes

**DOI:** 10.1155/2023/6305484

**Published:** 2023-03-29

**Authors:** Christin Körsten, Amira A. Al-Hosary, Cora M. Holicki, Mandy Schäfer, Birke A. Tews, Ana Vasić, Ute Ziegler, Martin H. Groschup, Cornelia Silaghi

**Affiliations:** ^1^Institute of Infectology, Friedrich-Loeffler Institute, Federal Research Institute for Animal Health, Greifswald 17493, Insel Riems, Germany; ^2^Department of Animal Medicine (Infectious Diseases), Faculty of Veterinary Medicine, Assiut University, Assiut 71526, Egypt; ^3^Institute of Novel and Emerging Infectious Diseases, Friedrich-Loeffler-Institute, Federal Research Institute for Animal Health, Greifswald 17493, Insel Riems, Germany; ^4^Scientific Institute of Veterinary Medicine of Serbia, Belgrade 11000, Serbia

## Abstract

The mosquito-borne zoonotic flaviviruses West Nile virus (WNV) and Usutu virus (USUV) are endemic in many European countries and emerged in Germany in recent years. Due to the increasing overlap of their distribution areas and their similar epidemiology, coinfections of WNV and USUV are possible. Indeed, coinfections in vertebrate hosts as a rare event have already been reported from some countries including Germany. However, it is largely unknown whether and to what extent coinfections could affect the vector competence of mosquitoes for WNV and USUV. For this purpose, the mosquito species *Culex pipiens* biotype *pipiens*, *Culex pipiens* biotype *molestus*, and *Aedes vexans* were orally infected in mono- and simultaneous coinfections with German strains of WNV and USUV. Mosquitoes were incubated for 14 days at 26°C, 85% relative humidity, and a 16 : 8 light-dark photocycle, before they were dissected and forced to salivate. The results showed a decrease in USUV susceptibility in *Culex pipiens* biotype *pipiens*, an increase in USUV susceptibility in *Aedes vexans*, and no obvious interaction between both viruses in *Culex pipiens* biotype *molestus*. Vector competence for WNV appeared to be unaffected by a simultaneous occurrence of USUV in all tested mosquito species. Coinfections with both viruses were only found in *Culex* mosquitoes, and cotransmission of WNV and USUV was observed in *Culex pipiens* biotype *molestus*. Overall, our results show that viral interactions between WNV and USUV vary between mosquito species, and that the interaction mainly occurs during infection and replication in the mosquito midgut. The results of this study confirm that to fully understand the interaction between WNV and USUV, studies with various mosquito species are necessary. In addition, we found that even mosquito species with a low susceptibility to both viruses, such as *Ae. vexans*, can play a role in their transmission in areas with cocirculation.

## 1. Introduction

In recent years, arboviruses have become an increasingly important threat to animal and human health in Europe. Due to climate change and globalization, mosquito-borne flaviviruses (genus *Flavivirus*, family *Flaviviridae*) such as West Nile virus (WNV) and Usutu virus (USUV) have spread throughout Europe and emerged in areas where they did not appear before [1–3].

In Germany, USUV was first detected in 2010 in *Culex* mosquitoes in the southwest of the country [4]. Since then there were repeated USUV outbreaks combined with massive death of birds, mostly blackbirds (*Turdus merula*) being affected [5–8]. To this date, USUV is distributed nationwide with several USUV lineages cocirculating in Germany [9–11]. In addition to mosquitoes and birds, viral RNA or neutralizing antibodies had also been detected in mammalian hosts such as horses [12, 13] and humans [14–16]. So far, no symptomatic human USUV infections have been reported in Germany, but the virus is known to also cause severe neurological diseases in individual cases [17, 18].

Several years after the emergence of USUV in Germany, WNV lineage 2 was first isolated from a great grey owl (*Strix nebulosa*) in 2018 in East Germany [19]. In the following years, a large number of WNV infections in birds and horses were reported [20], as well as infections in *Cx. pipiens* mosquitoes [21] and autochthonous infections in humans [16, 22, 23]. Detection of WNV RNA in hibernating *Cx. pipiens* in winter 2020/2021 confirmed the assumption that WNV can overwinter in mosquitoes in Germany [24]. Currently, WNV distribution is limited to the eastern part of Germany [11, 12]. As WNV can cause severe neurological diseases in different vertebrate hosts, it is a major threat to animal and human health [25–27].

There are many similarities between USUV and WNV. Both viruses have rapidly spread throughout Europe in recent years, resulting in their cocirculation in many European countries including Germany [11, 28–31]. In addition to the geographical overlap, WNV and USUV also share genetical and epidemiological characteristics. Both viruses are transmitted by mosquitoes as biological vectors in an enzootic cycle between mosquitoes and birds, but are also able to infect several other mammalian species such as humans or horses as dead-end hosts [28, 32, 33]. Due to their overlap in distribution areas, transmission cycles, vectors, and vertebrate hosts, coinfections of WNV and USUV can be expected and have to some extent already been detected in a human in Austria [29] and in birds in Italy and Germany [34, 35].

The main vector for both WNV and USUV are *Cx. pipiens* mosquitoes [28, 33], making this species complex the most susceptible to coinfections. The ability of German *Cx. pipiens* mosquitoes to transmit German WNV and USUV strains was confirmed experimentally in separate vector competence studies with field-collected as well as laboratory colonies [36, 37]. Infected *Cx. pipiens* were subsequently capable of experimentally infecting domestic geese with WNV through their bite, providing sound evidence for their possible role in the enzootic transmission cycle [38].

There are two forms of *Cx. pipiens* (biotype *pipiens* and *molestus*) that differ in their genetics and behavior [39, 40]. Biotype *pipiens* is considered to be a rather ornithophilic species that plays a particularly important role in the endemic cycles of the viruses, but can also transmit them to mammals as bridge vectors [41, 42]. Biotype *molestus* has a more mammalophilic feeding preference and often lives in urban areas near humans [42, 43], making it an important bridge vector for transmitting WNV and USUV to humans.

In addition to the *Cx. pipiens* biotypes, several other species are also considered to play a role in the transmission of both WNV and USUV. Potential candidates for coinfections are, amongst others: *Culex modestus, Aedes albopictus, Aedes cantans*, and *Aedes vexans* [37, 44–46]. *Aedes vexans* is one of the most frequent mosquito species with a high local abundance in Germany [41, 47]. Due to its mammalophilic feeding pattern, this species is certainly not the primary vector in the enzootic transmission of WNV and USUV compared to the *Cx. pipiens* biotypes [41]. However, the immature stages of this mosquito species are found in inundated areas such as floodplains of rivers and lakes, which are also natural habitats of many bird species, and *Ae. vexans* occasionally also feed on birds, especially when they occur in high abundance [48, 49]. In the event of an infection, *Ae. vexans* should not be underestimated as bridge vectors for WNV and USUV. So far, there have been no detections of WNV or USUV in field-collected*Ae. vexans* from Germany [21, 50], but in other countries WNV and USUV have been detected in this species reviewed in [44]. In addition, vector competence for WNV was proven for north American populations [51, 52]. However, to our best knowledge, the vector competence of *Ae. vexans* for USUV has not yet been investigated experimentally.

Despite the increasing importance of coinfections with WNV and USUV, knowledge of their impact on the vector competence of mosquitoes is largely unknown. Coinfections in mosquitoes could result in an increase or decrease of transmission of either or both viruses [53]. In addition, simultaneous transmission of both viruses to vertebrate hosts might be possible, which could have negative effects on the course of the infection [53, 54]. A study examining coinfections in *Cx. pipiens* biotype *pipiens* found a reduced vector competence for USUV in simultaneous coinfections as well as a reduced vector competence for WNV in case of a previous USUV infection [55]. However, it remains unclear whether and to what extent coinfections affect other mosquito species. Coinfection studies with other arboviruses revealed large variabilities with sometimes contradictory results, possibly due to differences in methodology and the used mosquito populations or virus strains [53]. Further evaluation of coinfections with WNV and USUV is therefore essential. In this study, we examined the effect of simultaneous coinfections with the same titers of WNV and USUV in *Cx. pipiens* biotype *pipiens*, *Cx. pipiens* biotype *molestus*, and *Ae. vexans*.

## 2. Material and Methods

### 2.1. Mosquito Origin and Rearing

Laboratory colonies of *Ae. vexans* “Green River” and *Cx. pipiens* biotype *molestus* originated from Utah, USA, in 2000 and from Hesse, Germany, in 2002, respectively. Both colonies were maintained in the laboratory ever since. For breeding, adult mosquitoes were offered bovine blood with the addition of heparin or EDTA twice a week. *Culex pipiens* biotype *molestus* egg rafts were collected in water filled beakers, while *Ae. vexans* laid their eggs into moist moss, that was subsequently stored at 10°C and flooded about 2.5 weeks before the infection experiment.

*Culex pipiens* biotype *pipiens* egg rafts were collected in June 2022 in Kleincarsdorf near Dresden, Germany, and species has been confirmed by using a multiplex RT-qPCR according to Rudolf et al. [56] with a pool of 10 larvae from each egg raft.

Larvae, pupae, and adult mosquitoes of all species were kept at 24–26°C, 60–85% relative humidity, and a 16 : 8 light-dark photocycle. Larvae were fed with ground Tetramin flakes (Tetra, Melle, Germany). Pupae were transferred into cages (Bugdorm; MegaView Science Co., Ltd., Taichung, Taiwan) to emerge. Adult mosquitoes for the infection experiments were offered 5% glucose ad libitum, but no blood. From each of the laboratory colonies, 12 individual mosquitoes were sampled and nucleic acids were extracted with the NucleoMag Vet Kit (Macherey-Nagel, Düren, Germany) according to the manufacturer's instructions in a KingFisher Flex (Thermo Fisher Scientific, Massachusetts, USA). From the *Cx. pipiens* biotype *pipiens*, a total of 68 adults were randomly selected, pooled into groups of 6–11 individuals and RNA was extracted using the Qiagen RNeasy Mini Kit (Qiagen, Hilden, Germany) according to the manufacturer's instructions. Presence of flavivirus RNA was tested with a RT-qPCR [57] to exclude any previous flavivirus infections.

### 2.2. Viruses and Cells

WNV lineage 2 “Germany 2018” (GenBank accession no. MH924836) was isolated from a great grey owl (*Strix nebulosa*) in Germany in 2018 [19]. The 4^th^ passage was propagated to a titer of 1.33 × 10^9^ 50% tissue culture infective dose per ml (TCID_50_/ml). USUV lineage Africa 3 (Genbank accession no. KY084496) was isolated from a blackbird (*Turdus merula*) in Germany in 2016 [58]. Two stocks of the 3^rd^ passage with a mean titer of 1.15 × 10^8^ TCID_50_/ml were produced.

Vero cells were obtained from the Biobank of the Friedrich-Loeffler Institut. Vero-76 cells were used for producing the USUV viral stocks. Vero-B4 cells were used for the propagation of WNV viral stock, titration of blood meals, and salivation assays.

### 2.3. Vector Competence Studies

**Figure 1:**
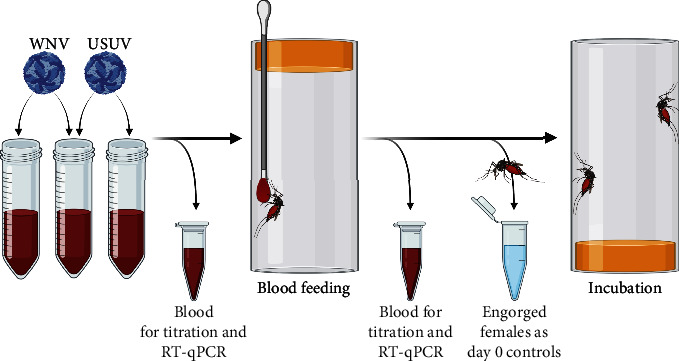
Procedure and sampling of mosquito infection. Created with https://BioRender.com.

Vector competence studies were performed similarly to those previously described [59]. The procedure of infection is shown in Figure 1.

One day prior to infection, female mosquitoes were sorted using a mouth aspirator with HEPA filter (John W. Hock Company, Florida, USA) for *Ae. vexans* or CO_2_ anesthesia for *Cx. pipiens* biotypes. Mosquitoes were placed in groups of approximately 10 individuals into plastic chambers closed with a sponge (50 mm × 100 mm; Carl Roth, Karlsruhe, Germany) without a sugar source. To minimize any additional intrinsic or extrinsic impacts on the vector competence, mono-and coinfections were done in temporal context (i.e., on the same day or on consecutive days) within the same mosquito population. In the case of *Ae. vexans* and *Cx. pipiens* biotype *molestus*, several infection trials were performed.

Virus stocks were mixed with heparinized bovine blood to a calculated blood meal titer of 1.00–1.78 × 10^7^ TCID_50_/ml for both viruses based on a recommendation from Vogels et al. [60]. In addition, 20 *μ*l of 5 mM adenosine triphosphate (Merck, Darmstadt, Germany) per 1 ml blood meal was added as phagostimulant. The infectious blood meal was immediately offered to the mosquitoes via soaked cotton sticks for 2-3 hours. At the beginning and at the end of feeding, blood meals containing only one virus were immediately titrated on Vero cells (seeded 1 × 10^5^ cells/ml the day before). Incubation of titration plates at 37°C and 5% CO_2_ was done for 7 days in an incubator (MCO-19AIC, Sanyo, Moriguchi, Japan). Subsequently, cells were stained and colored with a 1% crystal violet solution (Carl Roth) in 7.4% formaldehyde (Carl Roth) for 24 hours and then evaluated for cytopathic effect (CPE). In addition, 140 *μ*l of the blood without virus and of each blood meal (from the mono- and coinfections) were sampled into a 2 ml tube (Eppendorf, Hamburg, Germany) filled with 560 *μ*l AVL buffer with carrier RNA (Qiagen) and stored at −80°C until further analysis.

After feeding, mosquitoes were anesthetized with CO_2_ to sort engorged females into groups of approximately 5–10 individuals into incubation chambers (50 mm × 100 mm, Carl Roth). Chambers were modified by cutting out the bottom and replacing it with mosquito net for feeding and observation of the mosquitoes. During each infection experiment, one or two engorged females were taken out as day 0 controls and sampled as described. Subsequently, mosquitoes were homogenized using a TissueLyser II (Qiagen) at 30 Hz for 2 minutes and stored at −80°C.

**Figure 2:**
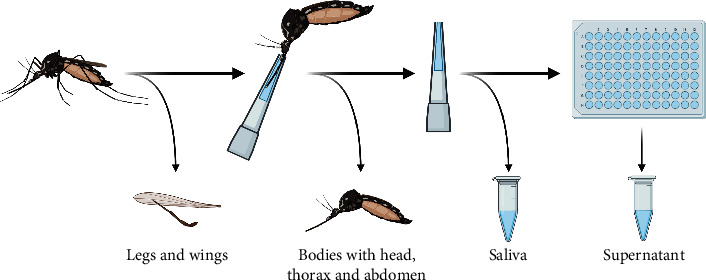
Dissection and forced salivation of survived mosquitoes. Created with https://BioRender.com.

Mosquitoes were incubated at 26°C, 85% relative humidity, and a 16 : 8 light-dark photocycle for 14 days in an incubator (MLR-352H-PE; Panasonic Corporation, Osaka, Japan). Feeding was done by placing a cotton pad soaked with 5% glucose on the mosquito net that closes the chambers. Fourteen days postinfection (dpi), surviving individuals were processed (Figure 2).

Mosquitoes were anesthetized with CO_2_ and immobilized by removing their legs and wings. Saliva samples were obtained as previously described [61]. Mosquito bodies, legs, and wings and approximately 10 *μ*l of the saliva samples were sampled as described above. Maceration of bodies and legs and wings was performed as described for the day 0 controls.

The remaining 10 *μ*l of the saliva samples were used to investigate for the presence of infectious virus (Figure 2). Therefore, a 96-well-plate was seeded with 1 × 10^5^ Vero cells/ml the day before. Before adding the saliva samples, culture medium was removed except for 20 *μ*l per well. Inoculated cells were incubated at 37°C for 1 hour before 200 *μ*l minimal essential medium with 2% fetal calf serum, 1% antibiotics (Gibco penicillin-streptomycin; Fisher Scientific, Schwerte, Germany), and 2% antimycotics (amphotericin B; Merck). Cells were incubated and stained as described for the blood meals. Before staining, cells were checked for CPE under a microscope (Nikon Eclipse Ts2, Nikon Europe B.V., Amstelveen, Netherlands), and 140 *μ*l supernatant of each well was sampled as described for the other samples.

### 2.4. Investigation for Viral RNA

All samples were stored at −80°C for at least 24 hours. After thawing, the samples were inactivated by incubation at 70°C for 10 minutes and centrifuged (Biofuge fresco; Heraeus instruments, Hanau, Germany) at 13000 rpm for 1 minute. 200 *μ*l supernatant was used for extraction with the NucleoMag Vet Kit (Macherey-Nagel) according to the manufacturer's instructions in a BioSprint 96 (Qiagen). For confirmation of successful extraction, 1 *μ*l internal control RNA [62] was added to each sample prior to automatic extraction.

For molecular investigation, a multiplex RT-qPCR was performed detecting the internal control RNA and either WNV or USUV RNA. For the detection of viral RNA, primers and a FAM-labelled probe targeting the WNV 5′ untranslated region [63] and the USUV NS1 region [4] were used. The internal control was detected with primers and a HEX-labelled probe was described by Hoffmann et al. [62]. RT-qPCR analysis was performed using the AgPath-ID kit (Thermo Fisher Scientific) in a CFX96 Real-Time PCR detection system (Bio-Rad Laboratories, Feldkirchen, Germany). Cross reactions between viral RNA assays and the respective other viruses were excluded by testing RNA extracts of the used viral stocks with both assays. For quantification, 10-fold dilution series of the used viral stocks (with known titers) were produced and extracted similarly to the mosquito samples. According to the results of the dilution series, a cut-off quantification cycle (Cq) value of 36.00 was determined for both PCR assays (Table S1).

### 2.5. Vector Competence Indices

In this study, the feeding rate is the percentage of engorged mosquitoes of the total number of living female mosquitoes that were offered an infectious blood meal. The survival rate is the percentage of living mosquitoes 14 dpi of all incubated mosquitoes.

The infection rate is defined as the percentage of mosquitoes with viral RNA in their bodies of all mosquitoes that survived until day 14 and is used as a measurement of susceptibility of the mosquito species. The dissemination rate is the percentage of positive legs and wings of the positive mosquito bodies. Legs and wings that contained viral RNA while the corresponding body was negative were considered negative. The transmission rate symbolizes the percentage of saliva containing viral RNA of all mosquitoes with a disseminated infection. Saliva samples were only considered positive when the corresponding body and legs and wings were also found positive, since a disseminated infection with viral replication in other tissue than the midgut is necessary to infect the salivary glands [64, 65]. The transmission efficiency is the percentage of positive saliva samples of all surviving mosquitoes and indicates the vector competence of a mosquito species. A transmission is assumed if the saliva sample contained viral RNA or if replicable virus could be isolated from the sample.

### 2.6. Statistical Analysis

The 95% confidence intervals of the vector competence indices were calculated using Microsoft Excel. Further statistical analysis and graphics were completed with SigmaPlot 11 (Systat Software, Düsseldorf, Germany). For comparison of feeding and survival rates, chi square tests were applied. For comparison of vector competence indices, Fisher's exact test was used. Comparison of two groups of viral loads was done with Student's *t*-test or Mann–Whitney Rank Sum test. For comparison of more than two groups, one-way ANOVA or Kruskal–Wallis test was performed. A statistical difference was assumed at a *p* value <0.05.

## 3. Results

### 3.1. Species Confirmation of Egg Rafts

Of the collected clutches of egg rafts, two were identified as *Cx. pipiens* biotype *pipiens* and three contained a mix of *Cx. pipiens* biotype *pipiens* and *Cx. torrentium*. Mosquitoes of the egg rafts containing both species that survived until day 14 were tested individually and were all confirmed as *Cx. pipiens* biotype *pipiens*.

### 3.2. Blood Meal Titers and Day 0 Samples

All blood samples without virus were found negative in RT-qPCR. The amount of viral RNA in equivalent of TCID_50_/ml in blood meal extracts did not agree with the titrated values and were therefore only used to confirm the addition of one or both viruses (Table S2).

In most experiments, blood meal titers of WNV and USUV in mono-infections varied between 3.65 × 10^6^ and 1.78 × 10^7^ TCID_50_/ml. No large decline in the virus titers during feeding was observed. For unknown reasons, a significantly higher titer than calculated were measured during a WNV mono-infection in *Ae. vexans* (8.66 × 10^7^ TCID_50_/ml). Since both titrations gave a high blood meal titer, probably too much viruses stock was accidentally used. However, since there were no significant differences in the amount of ingested virus in day 0 samples and the infection rates in this experiment compared to those in the other experiments, mosquitoes from this trial were not excluded from analysis. Apart from that aforementioned experiment there were no statistically significant differences between the blood meal titers of WNV and USUV (Table S2). In addition, there were no differences in the viral loads in day 0 samples between the viruses, mono- or coinfections and different mosquito species (Table S2).

### 3.3. Feeding and Survival Rates

For *Ae. vexans* and *Cx. pipiens* biotype *molestus*, no significant differences were found in feeding rates between the different viral infections (Table S3). However, in *Cx. pipiens* biotype *pipiens*, feeding rates in the mono-infections with WNV (28/65, 43.08%) and USUV (32/60, 53.33%) were both significantly higher (*p*=0.045; *p*=0.002) than the feeding rate for the coinfection (18/71, 25.35%). Overall, *Ae. vexans* showed a significantly higher feeding rate (183/307, 59.61%) than *Cx. pipiens* biotype *molestus* (319/924, 34.52%; *p* ≤ 0.001) and *Cx. pipiens* biotype *pipiens* (78/196, 39.80%; *p* ≤ 0.001).

Survival rates within *Cx. pipiens* biotype *pipiens* and *Cx. pipiens* biotype *molestus* were not significantly different for the distinct infections (Table S3). In *Ae. vexans*, the survival rate after mono-infection with WNV was significantly lower (26/62, 41.94%) than after USUV mono-infection (53/73, 72.60%; *p* ≤ 0.001). Overall, however, there was no evidence that simultaneous coinfections with WNV and USUV cause higher mortality rates in mosquitoes (Table S3).

### 3.4. Comparison of Vector Competences in Mono-Infections

In *Cx. pipiens* biotype *pipiens*, the dissemination rate of WNV (10/11, 90.90%) was significantly higher (*p*=0.023) than of USUV (9/19, 47.37%). However, the higher dissemination of WNV did not result in a higher transmission of WNV compared to USUV. Apart from this, there were no other statistically significant differences in the vector competence indices or viral loads in bodies, legs and wings, and saliva for WNV and USUV within the individual mosquito species (Tables S4–S6).

**Figure 3:**
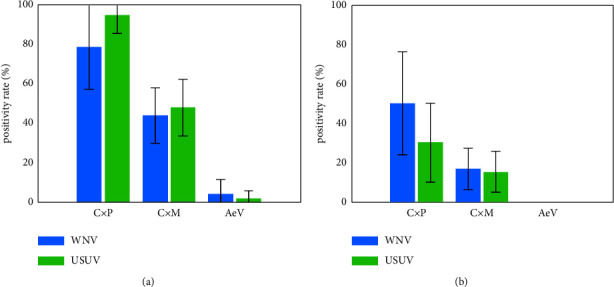
Comparison of vector competences of *Cx. pipiens* biotype *pipiens* (CxP), *Cx. pipiens* biotype *molestus* (CxM), and *Ae. vexans* (AeV) for WNV and USUV in mono-infections. (a) Infection rates in infected bodies per surviving mosquitoes. (b) Transmission efficiencies in saliva containing viral RNA per surviving mosquitoes. Error bars indicate 95% confidence interval.

Overall, we observed a high variability of vector competences for WNV and USUV between the three tested species (Figure 3, Tables S4–S6). Infection rates and transmission efficiencies of WNV and USUV in both *Culex* species were significantly higher than in *Ae. vexans* (Figure 3). In *Ae. vexans* we observed a very low susceptibility for both viruses and no transmission. *Culex pipiens* biotype *pipiens* and *Cx. pipiens* biotype *molestus* were both vector-competent for WNV and USUV, but *Cx. pipiens* biotype *pipiens* had significantly higher infection rates and transmission efficiencies than *Cx. pipiens* biotype *molestus*. Dissemination and transmission rates were comparable between *Cx. pipiens* biotype *pipiens* and *Cx. pipiens* biotype *molestus* (Tables S4 and S5). There were no statistical differences between the viral loads in bodies, legs and wings, and saliva samples of the different mosquito species (Tables S4–S6).

### 3.5. Impact of Co-Infections on the Vector Competence

Infection, dissemination, and transmission rates as well as transmission efficiencies of all tested species for WNV and USUV in mono-and coinfections are summarized in Table 1.

**Figure 4:**
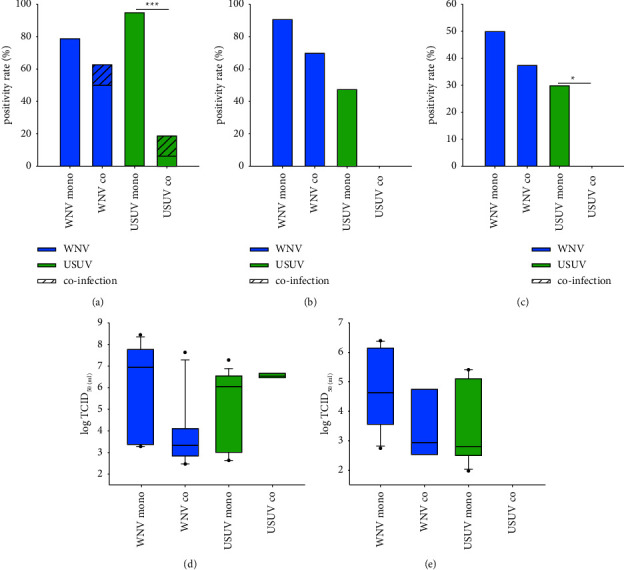
Coinfections with WNV and USUV reduce susceptibility to USUV in *Cx. pipiens* biotype *pipiens*. (a) Infection rates in infected mosquito bodies per surviving mosquitoes. (b) Dissemination rates in positive legs/wings per infected mosquito bodies. (c) Transmission efficiencies in saliva containing viral RNA per surviving mosquitoes. (d) Amount of viral RNA in equivalent to TCID_50_/ml in infected mosquito bodies. (e) Amount of viral RNA in equivalent to TCID_50_/ml in infected legs/wings. Stacked bar charts present the proportion of coinfected mosquito samples. (^*∗*^) and (^*∗∗∗*^) indicate statistically significant differences with *p* < 0.05 and *p* ≤ 0.001, respectively.

Susceptibility of *Cx. pipiens* biotype *pipiens* to USUV was significantly decreased in coinfection with WNV compared to mono-infections (Table 1 and Figure 4(a)). Consequently, USUV transmission efficiency was also reduced (Figure 4(c)). In contrast, differences of WNV vector competence were not statistically different, although vector competence indices and the amount of WNV RNA in infected mosquitoes appeared to be reduced in coinfections (Table S4 and Figures 4(d) and 4(e)). Two mosquito bodies were found to be coinfected with USUV and WNV, however, no codissemination or cotransmission was observed (Table S4 and Figures 4(a)–4(c)).

**Figure 5:**
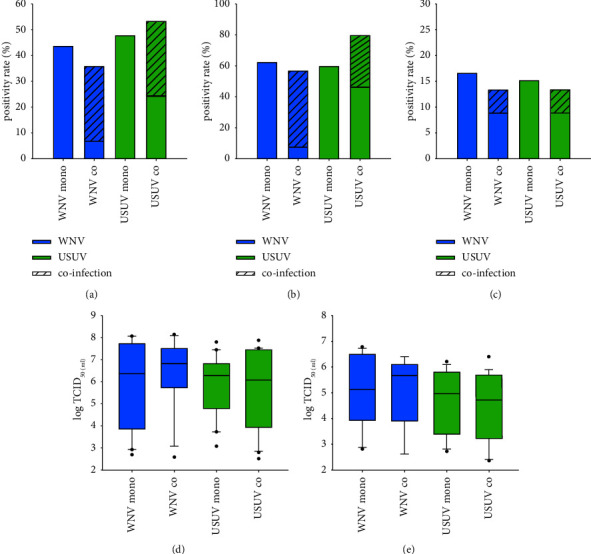
No obvious interaction between WNV and USUV in *Cx. pipiens* biotype *molestus*. (a) Infection rates in infected mosquito bodies per surviving mosquitoes. (b) Dissemination rates in positive legs/wings per infected mosquito bodies. (c) Transmission efficiencies in saliva containing viral RNA per surviving mosquitoes. (d) Amount of viral RNA in equivalent to TCID_50_/ml in infected mosquito bodies. (e) Amount of viral RNA in equivalent to TCID_50_/ml in infected legs/wings. Stacked bar charts present the proportion of coinfected mosquito samples.

In *Cx. pipiens* biotype *molestus*, no interaction between WNV and USUV was observed (Table 1 and Figure 5). Vector competence indices and viral loads in bodies, legs and wings and saliva of WNV and USUV were comparable between mono-and coinfections (Table 1, Table S5 and Figure 5). Coinfections with both viruses were frequently detected, and cotransmission was observed for two mosquitoes (Figures 5(a)–5(c)). Infectious particles of both viruses could be confirmed in these two saliva samples by salivation assay (Table S5). Interestingly, in coinfected mosquitoes, single dissemination of USUV occurred more frequently than a dissemination together with WNV, while WNV almost only disseminated together with USUV (Figure 5(b); *p*=0.039).

**Figure 6:**
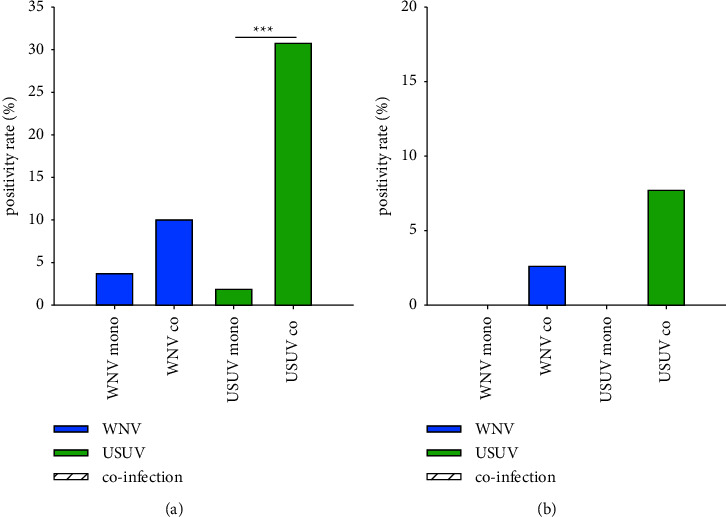
Coinfections with WNV and USUV increase susceptibility to USUV in *Ae. vexans*. (a) Infection rates in infected mosquito bodies per surviving mosquitoes. (b) Transmission efficiencies in saliva containing viral RNA per surviving mosquitoes. (^*∗∗∗*^) indicates a statistically significant difference with *p* ≤ 0.001. No sample was found positive for both viruses. Because of low positivity rates, analysis of other vector competence indices and viral loads were not done.

In *Ae. vexans*, susceptibility to USUV was significantly increased by a simultaneous occurrence of WNV (Table 1 and Figure 6(a)). By contrast, vector competence for WNV seemed not to be affected by a coinfection with USUV. Viral transmission was only observed in mosquitoes challenged with both viruses, but the differences were not statistically significant (Figure 6(b)). Potential differences in viral loads could not be investigated because of the low infection rates of these mosquitoes to WNV and USUV in the mono-infections. None of the *Ae. vexans* mosquitoes were found to be infected with both viruses.

## 4. Discussion

In this study, we investigated coinfections with WNV and USUV in three different vector species (*Cx. pipiens* biotype *pipiens*, *Cx. pipiens* biotype *molestus*, and *Ae. vexans*) to evaluate whether and to what extent both viruses interact in German mosquito species and how these interactions might influence the transmission of WNV and USUV. The results show that the interactions between the viruses and the mosquito vector can be species- and even biotype-dependent. While a simultaneous coinfection resulted in a reduction of the vector competence for USUV in *Cx. pipiens* biotype *pipiens*, the infection rate of *Ae. vexans* for USUV was increased. In *Cx. pipiens* biotype *molestus*, however, no change was observed in the vector competence, indicating no significant interaction between the viruses in this species.

When comparing the feeding rates in mono-and coinfections, a reduced feeding rate for *Cx. pipiens* biotype *pipiens* in the coinfection was observed compared to the feeding rates in the mono-infections. This may have been due to the higher proportion of medium in the infectious blood meal containing both viruses. However, we did not observe this effect for the other two species. Other studies that performed oral simultaneous coinfections in mosquitoes did not provide feeding rates, so comparison with other studies was not possible. Nevertheless, since the cause of the reduced feeding rate was probably the artificially produced infectious blood meal, this observation likely does not play a role in the actual feeding preference in nature.

For all three tested species, we did not detect any significant differences in their vector competences for WNV and USUV in mono-infections. This agrees with studies where a *Cx. pipiens* biotype *molestus* laboratory colony showed similar vector competences for WNV lineage 2 and USUV Africa 2 strains from Germany [36, 37]. In contrast, Fros et al. [66] found that *Cx. pipiens* mosquitoes were more effective vectors for an Italian USUV Europe 2 strain than for a WNV lineage 2 strain from Greece. Reasons for these differences could be the different viral strains that were used, or variations in methodology [60, 67].

In our study, both *Cx. pipiens* biotypes were found to be vector-competent for both WNV and USUV in mono-infections. *Cx. pipiens* biotype *pipiens* proved to be a more competent vector for WNV “Germany 2018” than biotype *molestus*, as it was already shown for the same viral strain in other populations [37] and also for other WNV strains [68]. Moreover, we found that *Cx. pipiens* biotype *pipiens* is also more competent for USUV than biotype *molestus*.

In contrast to the *Cx. pipiens* biotypes, *Ae. vexans* were not vector-competent for both WNV and USUV in mono-infections. Some *Ae. vexans* mosquitoes were exposed to a higher blood meal titer during a WNV mono-infection. It has already been shown that a higher viral dose can lead to an increased infection rate [36, 69]. However, this was not the case in the *Ae. vexans*, which were not susceptible to WNV even after exposure to a higher blood meal titer. This indicates that our chosen blood meal titer was not decisive for this result. In a previous study, the colony “Green River” was already tested for its vector competence for WNV lineages 1 and 2 [70]. Infection rates of *Ae. vexans* to WNV was higher in that study than in ours, possibly caused by usage of different WNV strains [60, 71]. Nevertheless, a transmission of WNV was not observed in either of the studies, indicating a low vector competence of *Ae. vexans* for WNV.

Although WNV and USUV cocirculate in many European countries [33], knowledge of the interaction of both viruses in mosquitoes is to date not sufficient. In a study by Wang et al. [55] a simultaneous coinfection with WNV lineage 2 and USUV Africa 3 in a laboratory colony of *Cx. pipiens* biotype *pipiens* led to a reduced vector competence for USUV. Using strains of the same WNV and USUV lineages, we were able to confirm this result for field-collected German *Cx. pipiens* biotype *pipiens*. Since our population was collected in an area near Dresden, where recently a coinfection of WNV and USUV in a small passerine bird was detected [35] these results can very well reflect the natural infection events in German mosquito populations. However, Wang et al. [55] also found that in a sequential infection, a previous USUV infection could reduce WNV vector competence in *Cx. pipiens* biotype *pipiens*. It remains unclear whether a sequential coinfection in German *Cx. pipiens* biotype *pipiens* would have the same outcome, and what impact this might have for the epidemiology of both viruses in Germany. In addition, there are multiple other USUV lineages circulating in Germany [11], and similar to vector competences for mono-infections, interaction of viruses in coinfections might differ between distinct virus lineages and strains [72].

In contrast to the obvious impact of coinfections on the vector competence of *Cx. pipiens* biotype *pipiens*, viral interaction in *Cx. pipiens* biotype *molestus* was not observed. Based on our results, interactions between WNV and USUV differ between the two *Cx. pipiens* biotypes. This is not surprising as both biotypes also vary in their vector competences as discussed above. *Culex pipiens* biotype *molestus* has a lower prevalence in Germany than biotype *pipiens* [56]. However, it often lives in urban regions [43], and therefore could transmit WNV and USUV to humans. Since no change in the transmission efficiencies of either virus in coinfected *Cx. pipiens* biotype *molestus* was observed, this study does not indicate the possibility of an altered transmission of WNV or USUV by this mosquito species in urban areas with cocirculation. In addition, we were for the first time able to demonstrate that coinfection and cotransmission of WNV and USUV in *Cx. pipiens* biotype *molestus* is possible.

Since our *Ae. vexans* were poorly susceptible to WNV and USUV in mono-infections, one might have assumed that no viral interaction would occur in this species. Interestingly, however, we observed an increased susceptibility to USUV in coinfections and, in contrast to the mono-infections, viral RNA was also found in saliva samples. These results indicate that even species with a low vector competence for WNV and USUV might play a greater role in their transmission in areas where both viruses cocirculate. Since our *Ae. vexans* colony originated from the United States, a transfer of the results to German *Ae. vexans* populations should be done with caution. Nevertheless, given the increased spread and cocirculation of WNV and USUV in Germany [11], mosquito species that have previously played little to no role in their transmission should also be considered for the monitoring and control of both viruses.

In *Cx. pipiens* biotype *pipiens* and *Ae. vexans*, the impact of coinfection on their vector competence was reflected in their infection rates. In order to infect a mosquito, arboviruses must overcome several barriers, and first infect the mosquito midgut after ingestion with an infectious blood meal [73]. The midgut barrier is considered the most important infection barrier and a significant factor for the vector competence of a mosquito species [74]. It is therefore not surprising that this barrier also plays a crucial role in the viral interaction during simultaneous coinfections. However, it remains unclear when and where viruses first start to interact after ingestion. As arboviruses initially only infect a few cells in the midgut [75], the interaction on a cellular level probably takes place in a later phase of replication in the midgut.

In *Cx. pipiens* biotype *pipiens*, which showed high susceptibility to both viruses in mono-infections, competition for suitable cellular receptors and cellular factors may have reduced the USUV infection rate, as both viruses are closely related and likely use the same receptors and cell components for their replication [76]. In *Ae. vexans*, the low susceptibility to WNV and USUV in mono-infections could have been an indication for the lack of cellular receptors used by these viruses [73]. However, the fact that increased infection with USUV was observed during coinfections suggests that suitable cellular receptors at least for USUV must be present in this species. Thus, not only the tissue barriers seem to play a role.

Another factor that has an impact on vector competence is the mosquito immune system. Mosquitoes do not have an adaptive immune system and cannot develop cross-immunity as in vertebrates [77]. The main antiviral pathway is the small interfering RNA (siRNA) within the RNA interference [78, 79]. It has already been shown that both WNV and USUV are targeted by siRNA in the mosquito vector [66]. Although USUV and WNV have similar genome sequences, there is no evidence of a cross reaction of siRNA against both viruses at the same time, and an increased reaction of siRNA in coinfections is therefore unlikely [55]. However, there is the possibility that in an event of simultaneous coinfection, the siRNA pathway is overwhelmed, making it easier for one or both viruses to overcome the mosquito immune system. In addition, flaviviruses produce a small subgenomic RNA during replication (sfRNA) [80] that is able to suppress the mosquito RNA interference pathway [81]. In case of a coinfection, the amount of sfRNA might also be increased, supporting the replication of one or both viruses. It is possible that in the case of *Ae. vexans*, USUV benefited from interactions with the mosquito immune system. Reasons as to why only USUV was enhanced are unclear and should be the subject to further investigations to get a better understanding of the interactions between WNV and USUV in the mosquito vector and in individual cells.

In summary, coinfections had an opposite effect on the USUV vector competence in *Cx. pipiens* biotype *pipiens* compared to *Ae. vexans*. Since no effects were observed in *Cx. pipiens* biotype *molestus*, interactions between WNV and USUV seem to be very dependent on the mosquito species. It is therefore necessary to test the interaction of both viruses in a broad range of mosquito species to get a better understanding of how and why WNV and USUV interact in mosquitoes.

## Figures and Tables

**Table 1:** Infection (IR), dissemination (DR), transmission rate (TR), and transmission efficiency (TE) for WNV lineage 2 and USUV Africa 3 in mono-and coinfections. (*n*/*n*) indicates absolute numbers.

Mosquito species	Virus infection		IR % (*n*/*n*)	DR % (*n*/*n*)	TR % (*n*/*n*)	TE % (*n*/*n*)
*Culex pipiens* biotype *pipiens*	WNV	Mono	78.57 (11/14)	90.90 (10/11)	70.00 (7/10)	50.00 (7/14)
		Co	62.50 (10/16)	70.00 (7/10)	85.71 (6/7)	37.50 (6/16)
	USUV	Mono	95.00 (19/20)	47.37 (9/19)	66.67 (6/9)	30.00 (6/20)
		Co	18.75 (3/16)	0.00 (0/3)	N/A	0.00 (0/16)

*Culex pipiens* biotype *molestus*	WNV	Mono	43.75 (21/48)	61.90 (13/21)	61.54 (8/13)	16.67 (8/48)
		Co	35.56 (16/45)	56.25 (9/16)	66.67 (6/9)	13.33 (6/45)
	USUV	Mono	47.83 (22/46)	59.09 (13/22)	53.85 (7/13)	15.22 (7/46)
		Co	53.33 (24/45)	79.17 (19/24)	31.58 (6/19)	13.33 (6/45)

*Aedes vexans*	WNV	Mono	3.85 (1/26)	0.00 (0/1)	N/A	0.00 (0/26)
		Co	10.26 (4/39)	75.00 (3/4)	33.33 (1/3)	2.56 (1/39)
	USUV	Mono	1.89 (1/53)	0.00 (0/1)	N/A	0.00 (0/53)
		Co	30.07 (12/39)	50.00 (6/12)	50.00 (3/6)	7.69 (3/39)

N/A, not applicable.

## Data Availability

The data that support the findings of this study are included within the main manuscript and in the supplementary material of this article.

## Supplementary Materials

Supplementary Materials Table S1: Results of 10-fold dilution series of viral stocks in RT-qPCR targeting USUV or WNV RNA. Table S2: Bloodmeal titers used in mono-and coinfections. Table S3: Feeding and survival rates of *Culex pipiens* biotype *pipiens, Culex pipiens* biotype *molestus*, and *Aedes vexans*. Table S4: Vector competence indices and viral loads in log TCID50/ml in *Culex pipiens* biotype *pipiens.* Table S5: Vector competence indices and viral loads in log TCID_50_/ml in *Culex pipiens* biotype *molestus*. Table S6: Vector competence indices and viral loads in log TCID_50_/ml in *Aedes vexans.*
