# Supplementary material for: Simultaneous Coinfections with West Nile Virus and Usutu Virus in Culex pipiens and Aedes vexans Mosquitoes
Source: Transbound Emerg Dis. 2023 Mar 29;2023:6305484. doi: 10.1155/2023/6305484 (PMC12016996; doi:10.1155/2023/6305484)
Supplement: Supplementary Materials — Table S1: Results of 10-fold dilution series of viral stocks in RT-qPCR targeting USUV or WNV RNA. Table S2: Bloodmeal titers used in mono-and coinfections. Table S3: Feeding and survival rates of Culex pipiens biotype pipiens, Culex pipiens biotype molestus, and Aedes vexans. Table S4: Vector competence indices and viral loads in log TCID50/ml in Culex pipiens biotype pipiens. Table S5: Vector competence indices and viral loads in log TCID50/ml in Culex pipiens biotype molestus. Table S6: Vector competence indices and viral loads in log TCID50/ml in Aedes vexans. [file 6305484.f1.docx]

**Table S1. Results of 10-fold dilution series of viral stocks in RT-qPCR targeting USUV or WNV RNA.**

| RT-qPCR Assay | Sample | Dilution | Titer in TCID_50_/ml | Cq | Cq 1^st^ repetition | Cq 2^nd^ repetition | Cq 3^rd^ repetition |
| --- | --- | --- | --- | --- | --- | --- | --- |
| USUV | USUV Africa 3 | -1 | 1.78E+07 | 18.28 | N/A | N/A | N/A |
|  | USUV Africa 3 | -2 | 1.78E+06 | 21.48 | 21.79 | N/A | N/A |
|  | USUV Africa 3 | -3 | 1.78E+05 | 25.08 | 25.05 | 25.14 | 25.09 |
|  | USUV Africa 3 | -4 | 1.78E+04 | 28.41 | 28.61 | 28.44 | 28.32 |
|  | USUV Africa 3 | -5 | 1.78E+03 | 31.71 | 32.16 | 31.68 | 31.86 |
|  | USUV Africa 3 | -6 | 1.78E+02 | 35.53 | 35.19 | 35.30 | 35.36 |
|  | USUV Africa 3 | -7 | 1.78E+01 | 39.25 | 37.88 | ND | ND |
|  | USUV Africa 3 | -8 | 1.78E+00 | 39.27 | ND | ND | ND |
|  | USUV Africa 3 | -9 | 1.78E-01 | ND | 39.19 | ND | ND |
|  | USUV Africa 3 | -10 | 1.78E-02 | ND | ND | ND | ND |
|  | USUV Africa 3 | -11 | 1.78E-03 | ND | ND | ND | ND |
|  | USUV Africa 3 | -12 | 1.78E-04 | ND | ND | ND | ND |
| WNV | WNV Germany 2018 | -1 | 1.33E+08 | 16.78 | 16.90 | N/A | N/A |
|  | WNV Germany 2018 | -2 | 1.33E+07 | 20.04 | 20.15 | N/A | N/A |
|  | WNV Germany 2018 | -3 | 1.33E+06 | 23.76 | 23.29 | 23.64 | 23.53 |
|  | WNV Germany 2018 | -4 | 1.33E+05 | 26.99 | 26.93 | 27.36 | 27.00 |
|  | WNV Germany 2018 | -5 | 1.33E+04 | 30.54 | 30.27 | 30.38 | 30.34 |
|  | WNV Germany 2018 | -6 | 1.33E+03 | 33.85 | 33.98 | 34.04 | 33.85 |
|  | WNV Germany 2018 | -7 | 1.33E+02 | 36.93 | 37.91 | 36.62 | 36.73 |
|  | WNV Germany 2018 | -8 | 1.33E+01 | 39.76 | 43.02 | 41.4 | ND |
|  | WNV Germany 2018 | -9 | 1.33E+00 | ND | ND | ND | ND |
|  | WNV Germany 2018 | -10 | 1.33E-01 | ND | ND | ND | ND |
|  | WNV Germany 2018 | -11 | 1.33E-02 | ND | ND | ND | ND |
|  | WNV Germany 2018 | -12 | 1.33E-03 | ND | ND | ND | ND |

N/A, not applicable
ND, not detected

**Table S2. Bloodmeal titers used in mono-and co-infections.**

|  |  |  |  | Bloodmeal 1 titer in log TCID_50_/ml | | Bloodmeal 2 titer in log TCID_50_/ml | | Amount of ingested virus in log TCID_50_/ml^a^ | |
| --- | --- | --- | --- | --- | --- | --- | --- | --- | --- |
| Species | Experiment no. | Virus | Infection | Titration | RT-qPCR^a^ | Titration | RT-qPCR^a^ | Engorged female 1 | Engorged female 2 |
| *Culex pipiens* biotype *pipiens* | #1 | USUV | mono | 6.875 | 3.539 | 7.250 | 3.518 | 4.473 | 3.902 |
|  |  | USUV | co | N/A | 0.49^b^ | N/A | 3.397 | 4.148 | 3.933 |
|  |  | WNV | mono | 7.125 | 4.563 | 7.125 | 4.499 | 4.472 | 4.769 |
|  |  | WNV | co | N/A | 4.375 | N/A | 4.738 | 4.780 | 4.623 |
| *Culex* *pipiens* biotype *molestus* | #1 | USUV | mono | 6.875 | 4.493 | 6.750 | 3.024 | N/A^c^ | N/A^c^ |
|  |  | USUV | co | N/A | 4.972 | N/A | 3.874 | 3.495 | 4.401 |
|  |  | WNV | mono | 6.750 | 5.262 | 6.938 | 4.817 | 4.743 | 4.074 |
|  |  | WNV | co | N/A | 5.208 | N/A | 4.991 | 3.800 | 4.748 |
|  | #2 | USUV | mono | 7.725 | 3.520 | 6.813 | 2.003 | 5.039 | N/A^c^ |
|  |  | USUV | co | N/A | 4.103 | N/A | 3.716 | 4.875 | N/A^c^ |
|  |  | WNV | mono | 7.000 | 5.210 | 7.250 | 4.881 | 5.464 | N/A^c^ |
|  |  | WNV | co | N/A | 5.225 | N/A | 4.861 | 5.117 | N/A^c^ |
|  | #3 | USUV | mono | 7.000 | 2.908 | 6.813 | 2.607 | 3.172 | ND^d^ |
|  |  | USUV | co | N/A | 3.178 | N/A | 2.034 | 4.265 | 3.927 |
|  |  | WNV | mono | 6.875 | 4.943 | 7.000 | 4.841 | 4.466 | 4.398 |
|  |  | WNV | co | N/A | 5.123 | N/A | 4.895 | 4.959 | 4.501 |
|  | #4 | USUV | mono | 7.063 | 4.372 | 6.563 | 2.519 | 3.462 | 3.850 |
|  |  | USUV | co | N/A | 4.414 | N/A | 3.963 | 4.508 | 4.325 |
|  |  | WNV | mono | 6.875 | 4.619 | 6.750 | 4.267 | 4.706 | 4.772 |
|  |  | WNV | co | N/A | 4.679 | N/A | 4.391 | 4.704 | 4.819 |
|  | #5 | USUV | mono | 6.875 | 3.539 | 7.250 | 3.518 | 4.329 | 4.231 |
|  |  | USUV | co | N/A | 0.49^b^ | N/A | 3.397 | 2.127 | 3.074 |
|  |  | WNV | mono | 7.125 | 4.563 | 7.125 | 4.499 | 4.395 | 4.515 |
|  |  | WNV | co | N/A | 4.375 | N/A | 4.738 | 3.395 | 3.883 |
| *Aedes vexans* | #1 | USUV | mono | 6.875 | 4.493 | 6.750 | 3.024 | 4.070 | 3.895 |
|  |  | USUV | co | N/A | 4.972 | N/A | 3.874 | 3.558 | 3.854 |
|  |  | WNV | mono | 6.750 | 5.262 | 6.938 | 4.817 | 4.629 | 4.479 |
|  |  | WNV | co | N/A | 5.208 | N/A | 4.991 | 3.774 | 4.103 |
|  | #2 | USUV | mono | 6.625 | 3.515 | 6.625 | 3.342 | 4.999 | 5.113 |
|  |  | USUV | co | N/A | 4.53 | N/A | 3.56 | 5.364 | 5.159 |
|  |  | WNV | mono | 7.875 | 6.067 | 8.000 | 6.037 | 6.132 | 5.998 |
|  |  | WNV | co | N/A | 5.287 | N/A | 5.199 | 5.677 | 5.507 |

N/A, not applicable

ND, not detected

^a^ Amount of viral RNA detected by RT-qPCR equivalent to log TCID_50_/ml

^b^ Since viral RNA was detected in the second sample, analysis was not repeated

^c^ Sample was not taken because of low number of engorged mosquitoes

^d^ Internal control RNA was detected, therefore an error during sampling is assumed

**Table S3. Feeding and survival rates of *Culex pipiens* biotype *pipiens, Culex pipiens* biotype *molestus* and *Aedes vexans*.**

| Species | Virus infection | Experiment  no. | Feeding rate % (n/n)^a^ | Survival rate % (n/n)^b^ |
| --- | --- | --- | --- | --- |
| *Culex  pipiens* biotype *pipiens*^c^ | USUV  mono-infection | #1 | 53.33 (32/60) | 56.67 (17/30) |
|  | WNV  mono-infection | #1 | 43.08 (28/65) | 50.00 (13/26) |
|  | co-infection | #1 | 25.35 (18/71) | 81.25 (13/16) |
| *Culex* *pipiens* biotype *molestus* | USUV  mono-infection | #1 | 3.17 (2/63)^d^ | 50.00 (1/2) |
|  |  | #2 | 44.00 (11/25) | 70.00 (7/10) |
|  |  | #3 | 27.37 (26/95) | 45.83 (11/24) |
|  |  | #4 | 48.91 (45/92) | 53.49 (23/43) |
|  |  | #5 | 18.75 (9/48) | 57.14 (4/7) |
|  | WNV  mono-infection | #1 | 29.85 (20/67) | 50.00 (9/18) |
|  |  | #2 | 22.22 (6/27) | 100.00 (5/5) |
|  |  | #3 | 24.24 (24/99) | 36.36 (8/22) |
|  |  | #4 | 47.96 (47/98) | 46.67 (21/45) |
|  |  | #5 | 40.38 (21/52) | 26.32 (5/19) |
|  | co-infection | #1 | 10.00 (7/70)^d^ | 20.00 (1/5) |
|  |  | #2 | 40.63 (13/32) | 66.67 (8/12) |
|  |  | #3 | 18.46 (24/130) | 50.00 (11/22) |
|  |  | #4 | 57.00 (57/100) | 36.36 (20/55) |
|  |  | #5 | 27.12 (16/59) | 35.71 (5/14) |
| *Aedes vexans* | USUV  mono-infection | #1 | 27.42 (17/62)^d^ | 46.67 (7/15) |
|  |  | #2 | 63.16 (60/95) | 79.31 (46/58) |
|  | WNV  mono-infection | #1 | 64.62 (42/65) | 32.50 (13/40) |
|  |  | #2 | 40.00 (24/60) | 59.09 (13/22) |
|  | co-infection | #1 | 19.70 (13/66)^d^ | 36.36 (4/11) |
|  |  | #2 | 65.52 (57/87) | 63.64 (35/55) |

^a^ Feeding rate in engorged females per living females on infection day

^b^ Survival rate in living females 14 days after infection per incubated females

^c^ Feeding and survival rate relate only to mosquitoes from egg rafts containing only *Culex pipiens* biotype *pipiens*, as dead mosquitoes were not tested for their species

^d^ Feeding rates were not included into analysis, because sorting was done one day after infection

**Table S4. Vector competence indices and viral loads in log TCID50/ml in *Culex pipiens* biotype *pipiens.***

| Virus | Virus  infection | Experiment no. | Mosquito  no. | Infection | Viral load^a^  in body | Dissemination | Viral load^a^  in legs/wings | Transmission | Viral load^a^  in saliva | Detection of infectious virus in saliva^b^ |
| --- | --- | --- | --- | --- | --- | --- | --- | --- | --- | --- |
| USUV | mono | #1 | #1 | infection | 6.099 | dissemination | 2.543 | no transmission | N/A | negative |
| USUV | mono | #1 | #2 | infection | 7.305 | dissemination | 5.433 | transmission | 3.15 | negative |
| USUV | mono | #1 | #3 | infection | 2.979 | dissemination | 2.506 | transmission | 3.9 | negative |
| USUV | mono | #1 | #4 | infection | 6.254 | no dissemination | N/A | N/A | N/A | negative |
| USUV | mono | #1 | #5 | infection | 6.316 | no dissemination | N/A | N/A | N/A | negative |
| USUV | mono | #1 | #6 | infection | 3.628 | dissemination | 2.813 | transmission | 2.503 | negative |
| USUV | mono | #1 | #7 | infection | 4.316 | no dissemination | N/A | N/A | N/A | negative |
| USUV | mono | #1 | #8 | infection | 3.325 | no dissemination | N/A | N/A | N/A | negative |
| USUV | mono | #1 | #9 | infection | 6.052 | no dissemination | N/A | N/A | N/A | negative |
| USUV | mono | #1 | #10 | infection | 2.774 | no dissemination | N/A | N/A | N/A | negative |
| USUV | mono | #1 | #11 | infection | 6.575 | dissemination | 2.492 | no transmission | N/A | negative |
| USUV | mono | #1 | #12 | no infection | N/A | N/A | N/A | N/A | N/A | negative |
| USUV | mono | #1 | #13 | infection | 2.646 | dissemination | 1.962 | transmission | 2.86 | negative |
| USUV | mono | #1 | #14 | infection | 6.906 | dissemination | 5.299 | transmission | 4.064 | positive (USUV) |
| USUV | mono | #1 | #15 | infection | 3.633 | dissemination | 2.883 | transmission | 2.596 | negative |
| USUV | mono | #1 | #16 | infection | 6.301 | dissemination | 2.974 | transmission | 2.563 | negative |
| USUV | mono | #1 | #17 | infection | 6.813 | dissemination | 5.059 | no transmission | N/A | negative |
| USUV | mono | #1 | #18 | infection | 6.798 | no dissemination | N/A | N/A | N/A | negative |
| USUV | mono | #1 | #19 | infection | 2.64 | no dissemination | N/A | N/A | N/A | negative |
| USUV | mono | #1 | #20 | infection | 2.885 | no dissemination | N/A | N/A | N/A | negative |
| USUV | co | #1 | #1 | no infection | N/A | N/A | N/A | N/A | N/A | negative |
| USUV | co | #1 | #2 | no infection | N/A | N/A | N/A | N/A | N/A | negative |
| USUV | co | #1 | #3 | no infection | N/A | N/A | N/A | N/A | N/A | negative |
| USUV | co | #1 | #4 | infection | 6.682 | no dissemination | N/A | N/A | N/A | negative |
| USUV | co | #1 | #5 | no infection | N/A | N/A | N/A | N/A | N/A | negative |
| USUV | co | #1 | #6 | no infection | N/A | N/A | N/A | N/A | N/A | negative |
| USUV | co | #1 | #7 | no infection | N/A | N/A | N/A | N/A | N/A | negative |
| USUV | co | #1 | #8 | no infection | N/A | N/A | N/A | N/A | N/A | negative |
| USUV | co | #1 | #9 | no infection | N/A | N/A | N/A | N/A | N/A | negative |
| USUV | co | #1 | #10 | no infection | N/A | N/A | N/A | N/A | N/A | negative |
| USUV | co | #1 | #11 | infection | 6.461 | no dissemination | N/A | N/A | N/A | negative |
| USUV | co | #1 | #12 | infection | 6.543 | no dissemination | N/A | N/A | N/A | negative |
| USUV | co | #1 | #13 | no infection | N/A | N/A | N/A | N/A | N/A | negative |
| USUV | co | #1 | #14 | no infection | N/A | N/A | N/A | N/A | N/A | negative |
| USUV | co | #1 | #15 | no infection | N/A | N/A | N/A | N/A | N/A | negative |
| USUV | co | #1 | #16 | no infection | N/A | N/A | N/A | N/A | N/A | negative |
| WNV | mono | #1 | #1 | no infection | N/A | N/A | N/A | N/A | N/A | negative |
| WNV | mono | #1 | #2 | infection | 7.419 | dissemination | 6.156 | transmission | 4.482 | negative |
| WNV | mono | #1 | #3 | infection | 3.297 | dissemination | 4.698 | no transmission | N/A | negative |
| WNV | mono | #1 | #4 | infection | 3.363 | dissemination | 2.747 | transmission | 2.91 | negative |
| WNV | mono | #1 | #5 | infection | 6.953 | dissemination | 3.822 | transmission | 3.131 | negative |
| WNV | mono | #1 | #6 | infection | 7.928 | dissemination | 5.795 | transmission | 4.669 | positive (WNV) |
| WNV | mono | #1 | #7 | infection | 3.443 | dissemination | 3.51 | no transmission | N/A | negative |
| WNV | mono | #1 | #8 | infection | 7.648 | dissemination | 6.205 | transmission | 3.538 | positive (WNV) |
| WNV | mono | #1 | #9 | infection | 8.478 | dissemination | 6.404 | transmission | 4.58 | positive (WNV) |
| WNV | mono | #1 | #10 | infection | 3.807 | dissemination | 4.539 | transmission | 3.219 | negative |
| WNV | mono | #1 | #11 | infection | 3.275 | dissemination | 3.564 | no transmission | N/A | negative |
| WNV | mono | #1 | #12 | infection | 7.796 | no dissemination | N/A | N/A | N/A | negative |
| WNV | mono | #1 | #13 | no infection | N/A | N/A | N/A | N/A | N/A | negative |
| WNV | mono | #1 | #14 | no infection | N/A | N/A | N/A | N/A | N/A | negative |
| WNV | co | #1 | #1 | infection | 2.854 | dissemination | 3.659 | transmission | 3.658 | negative |
| WNV | co | #1 | #2 | infection | 3.584 | dissemination | 2.561 | transmission | 3.149 | negative |
| WNV | co | #1 | #3 | no infection | N/A | N/A | N/A | N/A | N/A | negative |
| WNV | co | #1 | #4 | no infection | N/A | N/A | N/A | N/A | N/A | negative |
| WNV | co | #1 | #5 | no infection | N/A | N/A | N/A | N/A | N/A | negative |
| WNV | co | #1 | #6 | infection | 4.133 | dissemination | 2.477 | transmission | 2.879 | negative |
| WNV | co | #1 | #7 | no infection | N/A | N/A | N/A | N/A | N/A | negative |
| WNV | co | #1 | #8 | no infection | N/A | N/A | N/A | N/A | N/A | negative |
| WNV | co | #1 | #9 | infection | 3.851 | dissemination | 2.938 | no transmission | N/A | negative |
| WNV | co | #1 | #10 | infection | 3.064 | no dissemination | N/A | N/A | N/A | negative |
| WNV | co | #1 | #11 | infection | 7.661 | dissemination | 6.212 | transmission | 4.099 | positive (WNV) |
| WNV | co | #1 | #12 | infection | 4.098 | dissemination | 4.786 | transmission | 3.472 | negative |
| WNV | co | #1 | #13 | infection | 2.925 | dissemination | 2.521 | transmission | 2.605 | negative |
| WNV | co | #1 | #14 | infection | 2.445 | no dissemination | N/A | N/A | N/A | negative |
| WNV | co | #1 | #15 | no infection | N/A | N/A | N/A | N/A | N/A | negative |
| WNV | co | #1 | #16 | infection | 2.75 | no dissemination | N/A | N/A | N/A | negative |

N/A, not applicable

^a^ Amount of viral RNA detected by RT-qPCR equivalent to log TCID_50_/ml

^b^ Saliva samples were considered positive for infectious virus if a cytopathic effect was observed and viral RNA was detected in the supernatant

**Table S5. Vector competence indices and viral loads in log TCID_50_/ml in *Culex pipiens* biotype *molestus*.**

| Virus | Virus  infection | Experiment no. | Mosquito  no. | Infection | Viral load^a^  in body | Dissemination | Viral load^a^  in legs/wings | Transmission | Viral load^a^  in saliva | Detection of infectious virus in saliva^b^ |
| --- | --- | --- | --- | --- | --- | --- | --- | --- | --- | --- |
| USUV | mono | #1 | #1 | no infection | N/A | N/A | N/A | N/A | N/A | negative |
| USUV | mono | #2 | #2 | infection | 6.603 | no dissemination | N/A | N/A | N/A | negative |
| USUV | mono | #2 | #3 | no infection | N/A | N/A | N/A | N/A | N/A | negative |
| USUV | mono | #2 | #4 | no infection | N/A | N/A | N/A | N/A | N/A | negative |
| USUV | mono | #2 | #5 | no infection | N/A | N/A | N/A | N/A | N/A | negative |
| USUV | mono | #2 | #6 | infection | 5.096 | no dissemination | N/A | N/A | N/A | negative |
| USUV | mono | #2 | #7 | no infection | N/A | N/A | N/A | N/A | N/A | negative |
| USUV | mono | #2 | #8 | infection | 7.162 | dissemination | 5.462 | transmission | 3.714 | positive (USUV) |
| USUV | mono | #3 | #9 | no infection | N/A | N/A | N/A | N/A | N/A | negative |
| USUV | mono | #3 | #10 | no infection | N/A | N/A | N/A | N/A | N/A | negative |
| USUV | mono | #3 | #11 | infection | 7.780 | dissemination | 5.803 | transmission | 4.775 | positive (USUV) |
| USUV | mono | #3 | #12 | infection | 3.809 | dissemination | 5.801 | transmission | 2.403 | negative |
| USUV | mono | #3 | #13 | infection | 3.083 | dissemination | 2.730 | no transmission | N/A | negative |
| USUV | mono | #3 | #14 | infection | 7.014 | dissemination | 5.235 | transmission | 3.115 | negative |
| USUV | mono | #3 | #15 | no infection | N/A | N/A | N/A | N/A | N/A | negative |
| USUV | mono | #3 | #16 | no infection | N/A | N/A | N/A | N/A | N/A | negative |
| USUV | mono | #3 | #17 | no infection | N/A | N/A | N/A | N/A | N/A | negative |
| USUV | mono | #3 | #18 | infection | 6.641 | no dissemination | N/A | N/A | N/A | negative |
| USUV | mono | #3 | #19 | no infection | N/A | N/A | N/A | N/A | N/A | negative |
| USUV | mono | #4 | #20 | infection | 5.894 | no dissemination | N/A | N/A | N/A | negative |
| USUV | mono | #4 | #21 | infection | 5.854 | no dissemination | N/A | N/A | N/A | negative |
| USUV | mono | #4 | #22 | no infection | N/A | N/A | N/A | N/A | N/A | negative |
| USUV | mono | #4 | #23 | no infection | N/A | N/A | N/A | N/A | N/A | negative |
| USUV | mono | #4 | #24 | no infection | N/A | N/A | N/A | N/A | N/A | negative |
| USUV | mono | #4 | #25 | no infection | N/A | N/A | N/A | N/A | N/A | negative |
| USUV | mono | #4 | #26 | no infection | N/A | N/A | N/A | N/A | N/A | negative |
| USUV | mono | #4 | #27 | no infection | N/A | N/A | N/A | N/A | N/A | negative |
| USUV | mono | #4 | #28 | no infection | N/A | N/A | N/A | N/A | N/A | negative |
| USUV | mono | #4 | #29 | no infection | N/A | N/A | N/A | N/A | N/A | negative |
| USUV | mono | #4 | #30 | no infection | N/A | N/A | N/A | N/A | N/A | negative |
| USUV | mono | #4 | #31 | no infection | N/A | N/A | N/A | N/A | N/A | negative |
| USUV | mono | #4 | #32 | no infection | N/A | N/A | N/A | N/A | N/A | negative |
| USUV | mono | #4 | #33 | infection | 5.708 | no dissemination | N/A | N/A | N/A | negative |
| USUV | mono | #4 | #34 | infection | 6.162 | no dissemination | N/A | N/A | N/A | negative |
| USUV | mono | #4 | #35 | infection | 6.759 | no dissemination | N/A | N/A | N/A | negative |
| USUV | mono | #4 | #36 | infection | 5.794 | no dissemination | N/A | N/A | N/A | negative |
| USUV | mono | #4 | #37 | no infection | N/A | N/A | N/A | N/A | N/A | negative |
| USUV | mono | #4 | #38 | infection | 7.439 | dissemination | 6.207 | transmission | 3.726 | positive (USUV) |
| USUV | mono | #4 | #39 | no infection | N/A | N/A | N/A | N/A | N/A | negative |
| USUV | mono | #4 | #40 | infection | 6.385 | dissemination | 4.968 | no transmission | N/A | negative |
| USUV | mono | #4 | #41 | infection | 6.391 | dissemination | 4.051 | no transmission | N/A | negative |
| USUV | mono | #4 | #42 | infection | 6.475 | dissemination | 3.083 | no transmission | N/A | negative |
| USUV | mono | #5 | #43 | infection | 7.466 | dissemination | 5.95 | no transmission | N/A | negative |
| USUV | mono | #5 | #44 | infection | 3.718 | dissemination | 3.685 | transmission | 2.847 | negative |
| USUV | mono | #5 | #45 | infection | 3.886 | dissemination | 3.767 | no transmission | N/A | negative |
| USUV | mono | #5 | #46 | infection | 3.87 | dissemination | 2.948 | transmission | 3.149 | negative |
| USUV | co | #1 | #1 | infection | 6.492 | dissemination | 2.692 | no transmission | N/A | negative |
| USUV | co | #2 | #2 | no infection | N/A | N/A | N/A | N/A | N/A | negative |
| USUV | co | #2 | #3 | infection | 7.484 | dissemination | 5.743 | transmission | 3.706 | positive (WNV and USUV) |
| USUV | co | #2 | #4 | infection | 7.237 | dissemination | 5.681 | transmission | 4.151 | positive (USUV) |
| USUV | co | #2 | #5 | no infection | N/A | N/A | N/A | N/A | N/A | negative |
| USUV | co | #2 | #6 | infection | 6.009 | dissemination | 3.382 | transmission | 3.819 | positive (WNV and USUV) |
| USUV | co | #2 | #7 | infection | 3.048 | dissemination | 2.425 | no transmission | N/A | negative |
| USUV | co | #2 | #8 | no infection | N/A | N/A | N/A | N/A | N/A | negative |
| USUV | co | #2 | #9 | infection | 2.513 | dissemination | 2.516 | no transmission | N/A | negative |
| USUV | co | #3 | #10 | no infection | N/A | N/A | N/A | N/A | N/A | negative |
| USUV | co | #3 | #11 | no infection | N/A | N/A | N/A | N/A | N/A | negative |
| USUV | co | #3 | #12 | no infection | N/A | N/A | N/A | N/A | N/A | negative |
| USUV | co | #3 | #13 | no infection | N/A | N/A | N/A | N/A | N/A | negative |
| USUV | co | #3 | #14 | no infection | N/A | N/A | N/A | N/A | N/A | negative |
| USUV | co | #3 | #15 | infection | 5.834 | no dissemination | N/A | N/A | N/A | negative |
| USUV | co | #3 | #16 | infection | 6.454 | dissemination | 3.942 | no transmission | N/A | negative |
| USUV | co | #3 | #17 | infection | 7.866 | dissemination | 5.907 | transmission | 4.162 | positive (USUV) |
| USUV | co | #3 | #18 | infection | 2.791 | dissemination | 4.792 | no transmission | N/A | negative |
| USUV | co | #3 | #19 | infection | 6.106 | dissemination | 3.521 | no transmission | N/A | negative |
| USUV | co | #3 | #20 | infection | 7.514 | dissemination | 6.406 | no transmission | N/A | negative |
| USUV | co | #4 | #21 | no infection | N/A | N/A | N/A | N/A | N/A | negative |
| USUV | co | #4 | #22 | infection | 3.144 | no dissemination | N/A | N/A | N/A | negative |
| USUV | co | #4 | #23 | infection | 5.873 | no dissemination | N/A | N/A | N/A | negative |
| USUV | co | #4 | #24 | no infection | N/A | N/A | N/A | N/A | N/A | negative |
| USUV | co | #4 | #25 | no infection | N/A | N/A | N/A | N/A | N/A | negative |
| USUV | co | #4 | #26 | no infection | N/A | N/A | N/A | N/A | N/A | negative |
| USUV | co | #4 | #27 | infection | 5.853 | no dissemination | N/A | N/A | N/A | negative |
| USUV | co | #4 | #28 | no infection | N/A | N/A | N/A | N/A | N/A | negative |
| USUV | co | #4 | #29 | no infection | N/A | N/A | N/A | N/A | N/A | negative |
| USUV | co | #4 | #30 | infection | 7.493 | dissemination | 5.467 | transmission | 3.628 | positive (USUV) |
| USUV | co | #4 | #31 | infection | 7.505 | dissemination | 5.502 | transmission | 3.18 | positive (USUV) |
| USUV | co | #4 | #32 | infection | 6.42 | dissemination | 5.012 | no transmission | N/A | negative |
| USUV | co | #4 | #33 | infection | 7.539 | dissemination | 5.673 | no transmission | N/A | negative |
| USUV | co | #4 | #34 | infection | 3.963 | dissemination | 4.052 | no transmission | N/A | negative |
| USUV | co | #4 | #35 | no infection | N/A | N/A | N/A | N/A | N/A | negative |
| USUV | co | #4 | #36 | infection | 6.235 | dissemination | 3.232 | no transmission | N/A | negative |
| USUV | co | #4 | #37 | infection | 6.106 | dissemination | 4.73 | no transmission | N/A | negative |
| USUV | co | #4 | #38 | infection | 2.923 | no dissemination | N/A | N/A | N/A | negative |
| USUV | co | #4 | #39 | infection | 6.036 | dissemination | 2.368 | no transmission | N/A | negative |
| USUV | co | #4 | #40 | no infection | N/A | N/A | N/A | N/A | N/A | negative |
| USUV | co | #5 | #41 | no infection | N/A | N/A | N/A | N/A | N/A | negative |
| USUV | co | #5 | #42 | no infection | N/A | N/A | N/A | N/A | N/A | negative |
| USUV | co | #5 | #43 | no infection | N/A | N/A | N/A | N/A | N/A | negative |
| USUV | co | #5 | #44 | no infection | N/A | N/A | N/A | N/A | N/A | negative |
| USUV | co | #5 | #45 | no infection | N/A | N/A | N/A | N/A | N/A | negative |
| WNV | mono | #1 | #1 | no infection | N/A | N/A | N/A | N/A | N/A | negative |
| WNV | mono | #1 | #2 | no infection | N/A | N/A | N/A | N/A | N/A | negative |
| WNV | mono | #1 | #3 | no infection | N/A | N/A | N/A | N/A | N/A | negative |
| WNV | mono | #1 | #4 | no infection | N/A | N/A | N/A | N/A | N/A | negative |
| WNV | mono | #1 | #5 | no infection | N/A | N/A | N/A | N/A | N/A | negative |
| WNV | mono | #1 | #6 | no infection | N/A | N/A | N/A | N/A | N/A | negative |
| WNV | mono | #1 | #7 | infection | 7.594 | no dissemination | N/A | N/A | N/A | negative |
| WNV | mono | #1 | #8 | infection | 4.797 | no dissemination | N/A | N/A | N/A | negative |
| WNV | mono | #1 | #9 | infection | 3.721 | no dissemination | N/A | N/A | N/A | negative |
| WNV | mono | #2 | #10 | no infection | N/A | N/A | N/A | N/A | N/A | negative |
| WNV | mono | #2 | #11 | no infection | N/A | N/A | N/A | N/A | N/A | negative |
| WNV | mono | #2 | #12 | no infection | N/A | N/A | N/A | N/A | N/A | negative |
| WNV | mono | #2 | #13 | infection | 8.079 | dissemination | 6.639 | transmission | 5.557 | negative |
| WNV | mono | #2 | #14 | infection | 2.694 | dissemination | 4.560 | transmission | 4.238 | negative |
| WNV | mono | #3 | #15 | no infection | N/A | N/A | N/A | N/A | N/A | negative |
| WNV | mono | #3 | #16 | no infection | N/A | N/A | N/A | N/A | N/A | negative |
| WNV | mono | #3 | #17 | no infection | N/A | N/A | N/A | N/A | N/A | negative |
| WNV | mono | #3 | #18 | infection | 7.987 | dissemination | 6.195 | transmission | 5.163 | positive (WNV) |
| WNV | mono | #3 | #19 | no infection | N/A | N/A | N/A | N/A | N/A | negative |
| WNV | mono | #3 | #20 | no infection | N/A | N/A | N/A | N/A | N/A | negative |
| WNV | mono | #3 | #21 | infection | 2.918 | no dissemination | N/A | N/A | N/A | negative |
| WNV | mono | #3 | #22 | no infection | N/A | N/A | N/A | N/A | N/A | negative |
| WNV | mono | #4 | #23 | no infection | N/A | N/A | N/A | N/A | N/A | negative |
| WNV | mono | #4 | #24 | infection | 6.363 | no dissemination | N/A | N/A | N/A | negative |
| WNV | mono | #4 | #25 | infection | 7.142 | no dissemination | N/A | N/A | N/A | negative |
| WNV | mono | #4 | #26 | no infection | N/A | N/A | N/A | N/A | N/A | negative |
| WNV | mono | #4 | #27 | no infection | N/A | N/A | N/A | N/A | N/A | negative |
| WNV | mono | #4 | #28 | infection | 8.053 | dissemination | 6.654 | transmission | 3.636 | positive (WNV) |
| WNV | mono | #4 | #29 | no infection | N/A | N/A | N/A | N/A | N/A | negative |
| WNV | mono | #4 | #30 | infection | 5.364 | no dissemination | N/A | N/A | N/A | negative |
| WNV | mono | #4 | #31 | no infection | N/A | N/A | N/A | N/A | N/A | negative |
| WNV | mono | #4 | #32 | no infection | N/A | N/A | N/A | N/A | N/A | negative |
| WNV | mono | #4 | #33 | no infection | N/A | N/A | N/A | N/A | N/A | negative |
| WNV | mono | #4 | #34 | infection | 4.459 | no dissemination | N/A | N/A | N/A | negative |
| WNV | mono | #4 | #35 | no infection | N/A | N/A | N/A | N/A | N/A | negative |
| WNV | mono | #4 | #36 | infection | 7.414 | dissemination | 5.657 | no transmission | N/A | negative |
| WNV | mono | #4 | #37 | infection | 7.418 | dissemination | 3.433 | no transmission | N/A | negative |
| WNV | mono | #4 | #38 | infection | 6.816 | dissemination | 3.009 | no transmission | N/A | negative |
| WNV | mono | #4 | #39 | no infection | N/A | N/A | N/A | N/A | N/A | negative |
| WNV | mono | #4 | #40 | no infection | N/A | N/A | N/A | N/A | N/A | negative |
| WNV | mono | #4 | #41 | infection | 5.932 | dissemination | 2.812 | no transmission | N/A | negative |
| WNV | mono | #4 | #42 | infection | 8.028 | dissemination | 6.763 | transmission | 4.201 | positive (WNV) |
| WNV | mono | #4 | #43 | no infection | N/A | N/A | N/A | N/A | N/A | negative |
| WNV | mono | #5 | #44 | infection | 7.906 | dissemination | 6.379 | transmission | 3.286 | negative |
| WNV | mono | #5 | #45 | infection | 3.997 | dissemination | 4.403 | transmission | 3.294 | negative |
| WNV | mono | #5 | #46 | infection | 3.023 | dissemination | 5.136 | transmission | 3.525 | negative |
| WNV | mono | #5 | #47 | infection | 3.181 | dissemination | 5.142 | no transmission | N/A | negative |
| WNV | mono | #5 | #48 | no infection | N/A | N/A | N/A | N/A | N/A | negative |
| WNV co | co | #1 | #1 | infection | 6.99 | no dissemination | N/A | N/A | N/A | negative |
| WNV co | co | #2 | #2 | no infection | N/A | N/A | N/A | N/A | N/A | negative |
| WNV co | co | #2 | #3 | infection | 8.024 | dissemination | 6.240 | transmission | 3.412 | positive (WNV and USUV) |
| WNV co | co | #2 | #4 | no infection | N/A | N/A | N/A | N/A | N/A | negative |
| WNV co | co | #2 | #5 | no infection | N/A | N/A | N/A | N/A | N/A | negative |
| WNV co | co | #2 | #6 | infection | 7.406 | dissemination | 5.830 | transmission | 3.071 | positive (WNV and USUV) |
| WNV co | co | #2 | #7 | no infection | N/A | N/A | N/A | N/A | N/A | negative |
| WNV co | co | #2 | #8 | no infection | N/A | N/A | N/A | N/A | N/A | negative |
| WNV co | co | #2 | #9 | no infection | N/A | N/A | N/A | N/A | N/A | negative |
| WNV co | co | #3 | #10 | no infection | N/A | N/A | N/A | N/A | N/A | negative |
| WNV co | co | #3 | #11 | no infection | N/A | N/A | N/A | N/A | N/A | negative |
| WNV co | co | #3 | #12 | infection | 5.796 | no dissemination | N/A | N/A | N/A | negative |
| WNV co | co | #3 | #13 | no infection | N/A | N/A | N/A | N/A | N/A | negative |
| WNV co | co | #3 | #14 | no infection | N/A | N/A | N/A | N/A | N/A | negative |
| WNV co | co | #3 | #15 | no infection | N/A | N/A | N/A | N/A | N/A | negative |
| WNV co | co | #3 | #16 | infection | 8.149 | dissemination | 6.401 | transmission | 3.838 | positive (WNV) |
| WNV co | co | #3 | #17 | no infection | N/A | N/A | N/A | N/A | N/A | negative |
| WNV co | co | #3 | #18 | no infection | N/A | N/A | N/A | N/A | N/A | negative |
| WNV co | co | #3 | #19 | infection | 8.059 | dissemination | 4.551 | transmission | 5.660 | positive (WNV) |
| WNV co | co | #3 | #20 | no infection | N/A | N/A | N/A | N/A | N/A | negative |
| WNV co | co | #4 | #21 | no infection | N/A | N/A | N/A | N/A | N/A | negative |
| WNV co | co | #4 | #22 | infection | 4.191 | no dissemination | N/A | N/A | N/A | negative |
| WNV co | co | #4 | #23 | infection | 6.636 | no dissemination | N/A | N/A | N/A | negative |
| WNV co | co | #4 | #24 | no infection | N/A | N/A | N/A | N/A | N/A | negative |
| WNV co | co | #4 | #25 | no infection | N/A | N/A | N/A | N/A | N/A | negative |
| WNV co | co | #4 | #26 | no infection | N/A | N/A | N/A | N/A | N/A | negative |
| WNV co | co | #4 | #27 | infection | 5.736 | no dissemination | N/A | N/A | N/A | negative |
| WNV co | co | #4 | #28 | no infection | N/A | N/A | N/A | N/A | N/A | negative |
| WNV co | co | #4 | #29 | no infection | N/A | N/A | N/A | N/A | N/A | negative |
| WNV co | co | #4 | #30 | no infection | N/A | N/A | N/A | N/A | N/A | negative |
| WNV co | co | #4 | #31 | no infection | N/A | N/A | N/A | N/A | N/A | negative |
| WNV co | co | #4 | #32 | infection | 7.523 | dissemination | 6.022 | transmission | 2.678 | positive (WNV) |
| WNV co | co | #4 | #33 | infection | 6.828 | dissemination | 5.096 | no transmission | N/A | negative |
| WNV co | co | #4 | #34 | infection | 2.596 | dissemination | 3.216 | no transmission | N/A | negative |
| WNV co | co | #4 | #35 | no infection | N/A | N/A | N/A | N/A | N/A | negative |
| WNV co | co | #4 | #36 | infection | 6.761 | dissemination | 2.642 | no transmission | N/A | negative |
| WNV co | co | #4 | #37 | no infection | N/A | N/A | N/A | N/A | N/A | negative |
| WNV co | co | #4 | #38 | no infection | N/A | N/A | N/A | N/A | N/A | negative |
| WNV co | co | #4 | #39 | infection | 7.088 | no dissemination | N/A | N/A | N/A | negative |
| WNV co | co | #4 | #40 | no infection | N/A | N/A | N/A | N/A | N/A | negative |
| WNV co | co | #5 | #41 | no infection | N/A | N/A | N/A | N/A | N/A | negative |
| WNV co | co | #5 | #42 | no infection | N/A | N/A | N/A | N/A | N/A | negative |
| WNV co | co | #5 | #43 | infection | 3.391 | dissemination | 5.671 | transmission | 3.33 | negative |
| WNV co | co | #5 | #44 | infection | 6.913 | no dissemination | N/A | N/A | N/A | negative |
| WNV co | co | #5 | #45 | no infection | N/A | N/A | N/A | N/A | N/A | negative |

N/A, not applicable

^a^ Amount of viral RNA detected by RT-qPCR equivalent to log TCID_50_/ml

^b^ Saliva samples were considered positive for infectious virus if a cytopathic effect was observed and viral RNA was detected in the supernatant

**Table S6. Vector competence indices and viral loads in log TCID_50_/ml in *Aedes vexans.***

| Virus | Virus  infection | Experiment no. | Mosquito  no. | Infection | Viral load^a^  in body | Dissemination | Viral load^a^  in legs/wings | Transmission | Viral load^a^  in saliva | Detection of infectious virus in saliva^b^ |
| --- | --- | --- | --- | --- | --- | --- | --- | --- | --- | --- |
| USUV | mono | #1 | #1 | no infection | N/A | N/A | N/A | N/A | N/A | negative |
| USUV | mono | #1 | #2 | no infection | N/A | N/A | N/A | N/A | N/A | negative |
| USUV | mono | #1 | #3 | no infection | N/A | N/A | N/A | N/A | N/A | negative |
| USUV | mono | #1 | #4 | no infection | N/A | N/A | N/A | N/A | N/A | negative |
| USUV | mono | #1 | #5 | no infection | N/A | N/A | N/A | N/A | N/A | negative |
| USUV | mono | #1 | #6 | no infection | N/A | N/A | N/A | N/A | N/A | negative |
| USUV | mono | #1 | #7 | no infection | N/A | N/A | N/A | N/A | N/A | negative |
| USUV | mono | #2 | #8 | no infection | N/A | N/A | N/A | N/A | N/A | negative |
| USUV | mono | #2 | #9 | no infection | N/A | N/A | N/A | N/A | N/A | negative |
| USUV | mono | #2 | #10 | no infection | N/A | N/A | N/A | N/A | N/A | negative |
| USUV | mono | #2 | #11 | no infection | N/A | N/A | N/A | N/A | N/A | negative |
| USUV | mono | #2 | #12 | no infection | N/A | N/A | N/A | N/A | N/A | negative |
| USUV | mono | #2 | #13 | no infection | N/A | N/A | N/A | N/A | N/A | negative |
| USUV | mono | #2 | #14 | no infection | N/A | N/A | N/A | N/A | N/A | negative |
| USUV | mono | #2 | #15 | no infection | N/A | N/A | N/A | N/A | N/A | negative |
| USUV | mono | #2 | #16 | no infection | N/A | N/A | N/A | N/A | N/A | negative |
| USUV | mono | #2 | #17 | no infection | N/A | N/A | N/A | N/A | N/A | negative |
| USUV | mono | #2 | #18 | no infection | N/A | N/A | N/A | N/A | N/A | negative |
| USUV | mono | #2 | #19 | no infection | N/A | N/A | N/A | N/A | N/A | negative |
| USUV | mono | #2 | #20 | no infection | N/A | N/A | N/A | N/A | N/A | negative |
| USUV | mono | #2 | #21 | no infection | N/A | N/A | N/A | N/A | N/A | negative |
| USUV | mono | #2 | #22 | no infection | N/A | N/A | N/A | N/A | N/A | negative |
| USUV | mono | #2 | #23 | no infection | N/A | N/A | N/A | N/A | N/A | negative |
| USUV | mono | #2 | #24 | no infection | N/A | N/A | N/A | N/A | N/A | negative |
| USUV | mono | #2 | #25 | no infection | N/A | N/A | N/A | N/A | N/A | negative |
| USUV | mono | #2 | #26 | no infection | N/A | N/A | N/A | N/A | N/A | negative |
| USUV | mono | #2 | #27 | no infection | N/A | N/A | N/A | N/A | N/A | negative |
| USUV | mono | #2 | #28 | no infection | N/A | N/A | N/A | N/A | N/A | negative |
| USUV | mono | #2 | #29 | no infection | N/A | N/A | N/A | N/A | N/A | negative |
| USUV | mono | #2 | #30 | no infection | N/A | N/A | N/A | N/A | N/A | negative |
| USUV | mono | #2 | #31 | no infection | N/A | N/A | N/A | N/A | N/A | negative |
| USUV | mono | #2 | #32 | no infection | N/A | N/A | N/A | N/A | N/A | negative |
| USUV | mono | #2 | #33 | no infection | N/A | N/A | N/A | N/A | N/A | negative |
| USUV | mono | #2 | #34 | no infection | N/A | N/A | N/A | N/A | N/A | negative |
| USUV | mono | #2 | #35 | no infection | N/A | N/A | N/A | N/A | N/A | negative |
| USUV | mono | #2 | #36 | no infection | N/A | N/A | N/A | N/A | N/A | negative |
| USUV | mono | #2 | #37 | no infection | N/A | N/A | N/A | N/A | N/A | negative |
| USUV | mono | #2 | #38 | no infection | N/A | N/A | N/A | N/A | N/A | negative |
| USUV | mono | #2 | #39 | no infection | N/A | N/A | N/A | N/A | N/A | negative |
| USUV | mono | #2 | #40 | no infection | N/A | N/A | N/A | N/A | N/A | negative |
| USUV | mono | #2 | #41 | no infection | N/A | N/A | N/A | N/A | N/A | negative |
| USUV | mono | #2 | #42 | infection | 3.183 | no dissemination | N/A | N/A | N/A | negative |
| USUV | mono | #2 | #43 | no infection | N/A | N/A | N/A | N/A | N/A | negative |
| USUV | mono | #2 | #44 | no infection | N/A | N/A | N/A | N/A | N/A | negative |
| USUV | mono | #2 | #45 | no infection | N/A | N/A | N/A | N/A | N/A | negative |
| USUV | mono | #2 | #46 | no infection | N/A | N/A | N/A | N/A | N/A | negative |
| USUV | mono | #2 | #47 | no infection | N/A | N/A | N/A | N/A | N/A | negative |
| USUV | mono | #2 | #48 | no infection | N/A | N/A | N/A | N/A | N/A | negative |
| USUV | mono | #2 | #49 | no infection | N/A | N/A | N/A | N/A | N/A | negative |
| USUV | mono | #2 | #50 | no infection | N/A | N/A | N/A | N/A | N/A | negative |
| USUV | mono | #2 | #51 | no infection | N/A | N/A | N/A | N/A | N/A | negative |
| USUV | mono | #2 | #52 | no infection | N/A | N/A | N/A | N/A | N/A | negative |
| USUV | mono | #2 | #53 | no infection | N/A | N/A | N/A | N/A | N/A | negative |
| USUV | co | #1 | #1 | no infection | N/A | N/A | N/A | N/A | N/A | negative |
| USUV | co | #1 | #2 | no infection | N/A | N/A | N/A | N/A | N/A | negative |
| USUV | co | #1 | #3 | no infection | N/A | N/A | N/A | N/A | N/A | negative |
| USUV | co | #1 | #4 | no infection | N/A | N/A | N/A | N/A | N/A | negative |
| USUV | co | #2 | #5 | no infection | N/A | N/A | N/A | N/A | N/A | negative |
| USUV | co | #2 | #6 | no infection | N/A | N/A | N/A | N/A | N/A | negative |
| USUV | co | #2 | #7 | no infection | N/A | N/A | N/A | N/A | N/A | negative |
| USUV | co | #2 | #8 | no infection | N/A | N/A | N/A | N/A | N/A | negative |
| USUV | co | #2 | #9 | no infection | N/A | N/A | N/A | N/A | N/A | negative |
| USUV | co | #2 | #10 | no infection | N/A | N/A | N/A | N/A | N/A | negative |
| USUV | co | #2 | #11 | no infection | N/A | N/A | N/A | N/A | N/A | negative |
| USUV | co | #2 | #12 | no infection | N/A | N/A | N/A | N/A | N/A | negative |
| USUV | co | #2 | #13 | no infection | N/A | N/A | N/A | N/A | N/A | negative |
| USUV | co | #2 | #14 | no infection | N/A | N/A | N/A | N/A | N/A | negative |
| USUV | co | #2 | #15 | no infection | N/A | N/A | N/A | N/A | N/A | negative |
| USUV | co | #2 | #16 | no infection | N/A | N/A | N/A | N/A | N/A | negative |
| USUV | co | #2 | #17 | infection | 3.885 | no dissemination | N/A | N/A | N/A | negative |
| USUV | co | #2 | #18 | no infection | N/A | N/A | N/A | N/A | N/A | negative |
| USUV | co | #2 | #19 | no infection | N/A | N/A | N/A | N/A | N/A | negative |
| USUV | co | #2 | #20 | no infection | N/A | N/A | N/A | N/A | N/A | negative |
| USUV | co | #2 | #21 | no infection | N/A | N/A | N/A | N/A | N/A | negative |
| USUV | co | #2 | #22 | no infection | N/A | N/A | N/A | N/A | N/A | negative |
| USUV | co | #2 | #23 | no infection | N/A | N/A | N/A | N/A | N/A | negative |
| USUV | co | #2 | #24 | no infection | N/A | N/A | N/A | N/A | N/A | negative |
| USUV | co | #2 | #25 | no infection | N/A | N/A | N/A | N/A | N/A | negative |
| USUV | co | #2 | #26 | infection | 6.924 | dissemination | 5.837 | no transmission | N/A | negative |
| USUV | co | #2 | #27 | infection | 3.692 | dissemination | 4.972 | transmission | 2.63 | negative |
| USUV | co | #2 | #28 | infection | 4.953 | dissemination | 4.035 | no transmission | N/A | negative |
| USUV | co | #2 | #29 | infection | 3.389 | dissemination | 4.643 | no transmission | N/A | negative |
| USUV | co | #2 | #30 | infection | 2.492 | dissemination | 3.512 | transmission | 4.483 | negative |
| USUV | co | #2 | #31 | infection | 3.224 | dissemination | 3.009 | transmission | 3.457 | negative |
| USUV | co | #2 | #32 | infection | 3.667 | no dissemination | N/A | N/A | N/A | negative |
| USUV | co | #2 | #33 | infection | 2.624 | no dissemination | N/A | N/A | N/A | negative |
| USUV | co | #2 | #34 | infection | 3.107 | no dissemination | N/A | N/A | N/A | negative |
| USUV | co | #2 | #35 | no infection | N/A | N/A | N/A | N/A | N/A | negative |
| USUV | co | #2 | #36 | no infection | N/A | N/A | N/A | N/A | N/A | negative |
| USUV | co | #2 | #37 | infection | 2.882 | no dissemination | N/A | N/A | N/A | negative |
| USUV | co | #2 | #38 | infection | 3.451 | no dissemination | N/A | N/A | N/A | negative |
| USUV | co | #2 | #39 | no infection | N/A | N/A | N/A | N/A | N/A | negative |
| WNV | mono | #1 | #1 | no infection | N/A | N/A | N/A | N/A | N/A | negative |
| WNV | mono | #1 | #2 | no infection | N/A | N/A | N/A | N/A | N/A | negative |
| WNV | mono | #1 | #3 | no infection | N/A | N/A | N/A | N/A | N/A | negative |
| WNV | mono | #1 | #4 | no infection | N/A | N/A | N/A | N/A | N/A | negative |
| WNV | mono | #1 | #5 | no infection | N/A | N/A | N/A | N/A | N/A | negative |
| WNV | mono | #1 | #6 | no infection | N/A | N/A | N/A | N/A | N/A | negative |
| WNV | mono | #1 | #7 | no infection | N/A | N/A | N/A | N/A | N/A | negative |
| WNV | mono | #1 | #8 | no infection | N/A | N/A | N/A | N/A | N/A | negative |
| WNV | mono | #1 | #9 | no infection | N/A | N/A | N/A | N/A | N/A | negative |
| WNV | mono | #1 | #10 | no infection | N/A | N/A | N/A | N/A | N/A | negative |
| WNV | mono | #1 | #11 | no infection | N/A | N/A | N/A | N/A | N/A | negative |
| WNV | mono | #1 | #12 | no infection | N/A | N/A | N/A | N/A | N/A | negative |
| WNV | mono | #1 | #13 | no infection | N/A | N/A | N/A | N/A | N/A | negative |
| WNV | mono | #2 | #14 | no infection | N/A | N/A | N/A | N/A | N/A | negative |
| WNV | mono | #2 | #15 | no infection | N/A | N/A | N/A | N/A | N/A | negative |
| WNV | mono | #2 | #16 | no infection | N/A | N/A | N/A | N/A | N/A | negative |
| WNV | mono | #2 | #17 | no infection | N/A | N/A | N/A | N/A | N/A | negative |
| WNV | mono | #2 | #18 | no infection | N/A | N/A | N/A | N/A | N/A | negative |
| WNV | mono | #2 | #19 | no infection | N/A | N/A | N/A | N/A | N/A | negative |
| WNV | mono | #2 | #20 | no infection | N/A | N/A | N/A | N/A | N/A | negative |
| WNV | mono | #2 | #21 | no infection | N/A | N/A | N/A | N/A | N/A | negative |
| WNV | mono | #2 | #22 | infection | 7.056 | no dissemination | N/A | N/A | N/A | negative |
| WNV | mono | #2 | #23 | no infection | N/A | N/A | N/A | N/A | N/A | negative |
| WNV | mono | #2 | #24 | no infection | N/A | N/A | N/A | N/A | N/A | negative |
| WNV | mono | #2 | #25 | no infection | N/A | N/A | N/A | N/A | N/A | negative |
| WNV | mono | #2 | #26 | no infection | N/A | N/A | N/A | N/A | N/A | negative |
| WNV | co | #1 | #1 | infection | 4.859 | dissemination | 3.087 | transmission | 4.015 | negative |
| WNV | co | #1 | #2 | infection | 3.694 | dissemination | 3.57 | no transmission | N/A | negative |
| WNV | co | #1 | #3 | infection | 3.255 | dissemination | 3.089 | no transmission | N/A | negative |
| WNV | co | #1 | #4 | infection | 3.259 | no dissemination | N/A | N/A | N/A | negative |
| WNV | co | #2 | #5 | no infection | N/A | N/A | N/A | N/A | N/A | negative |
| WNV | co | #2 | #6 | no infection | N/A | N/A | N/A | N/A | N/A | negative |
| WNV | co | #2 | #7 | no infection | N/A | N/A | N/A | N/A | N/A | negative |
| WNV | co | #2 | #8 | no infection | N/A | N/A | N/A | N/A | N/A | negative |
| WNV | co | #2 | #9 | no infection | N/A | N/A | N/A | N/A | N/A | negative |
| WNV | co | #2 | #10 | no infection | N/A | N/A | N/A | N/A | N/A | negative |
| WNV | co | #2 | #11 | no infection | N/A | N/A | N/A | N/A | N/A | negative |
| WNV | co | #2 | #12 | no infection | N/A | N/A | N/A | N/A | N/A | negative |
| WNV | co | #2 | #13 | no infection | N/A | N/A | N/A | N/A | N/A | negative |
| WNV | co | #2 | #14 | no infection | N/A | N/A | N/A | N/A | N/A | negative |
| WNV | co | #2 | #15 | no infection | N/A | N/A | N/A | N/A | N/A | negative |
| WNV | co | #2 | #16 | no infection | N/A | N/A | N/A | N/A | N/A | negative |
| WNV | co | #2 | #17 | no infection | N/A | N/A | N/A | N/A | N/A | negative |
| WNV | co | #2 | #18 | no infection | N/A | N/A | N/A | N/A | N/A | negative |
| WNV | co | #2 | #19 | no infection | N/A | N/A | N/A | N/A | N/A | negative |
| WNV | co | #2 | #20 | no infection | N/A | N/A | N/A | N/A | N/A | negative |
| WNV | co | #2 | #21 | no infection | N/A | N/A | N/A | N/A | N/A | negative |
| WNV | co | #2 | #22 | no infection | N/A | N/A | N/A | N/A | N/A | negative |
| WNV | co | #2 | #23 | no infection | N/A | N/A | N/A | N/A | N/A | negative |
| WNV | co | #2 | #24 | no infection | N/A | N/A | N/A | N/A | N/A | negative |
| WNV | co | #2 | #25 | no infection | N/A | N/A | N/A | N/A | N/A | negative |
| WNV | co | #2 | #26 | no infection | N/A | N/A | N/A | N/A | N/A | negative |
| WNV | co | #2 | #27 | no infection | N/A | N/A | N/A | N/A | N/A | negative |
| WNV | co | #2 | #28 | no infection | N/A | N/A | N/A | N/A | N/A | negative |
| WNV | co | #2 | #29 | no infection | N/A | N/A | N/A | N/A | N/A | negative |
| WNV | co | #2 | #30 | no infection | N/A | N/A | N/A | N/A | N/A | negative |
| WNV | co | #2 | #31 | no infection | N/A | N/A | N/A | N/A | N/A | negative |
| WNV | co | #2 | #32 | no infection | N/A | N/A | N/A | N/A | N/A | negative |
| WNV | co | #2 | #33 | no infection | N/A | N/A | N/A | N/A | N/A | negative |
| WNV | co | #2 | #34 | no infection | N/A | N/A | N/A | N/A | N/A | negative |
| WNV | co | #2 | #35 | no infection | N/A | N/A | N/A | N/A | N/A | negative |
| WNV | co | #2 | #36 | no infection | N/A | N/A | N/A | N/A | N/A | negative |
| WNV | co | #2 | #37 | no infection | N/A | N/A | N/A | N/A | N/A | negative |
| WNV | co | #2 | #38 | no infection | N/A | N/A | N/A | N/A | N/A | negative |
| WNV | co | #2 | #39 | no infection | N/A | N/A | N/A | N/A | N/A | negative |

N/A, not applicable

^a^ Amount of viral RNA detected by RT-qPCR equivalent to log TCID_50_/ml

^b^ Saliva samples were considered positive for infectious virus if a cytopathic effect was observed and viral RNA was detected in the supernatant
